# Expression of *De Novo* Open Reading Frames in Natural Populations of *Drosophila melanogaster*

**DOI:** 10.1002/jez.b.23297

**Published:** 2025-04-15

**Authors:** Amanda Glaser‐Schmitt, Marie Lebherz, Ezgi Saydam, Erich Bornberg‐Bauer, John Parsch

**Affiliations:** ^1^ Division of Evolutionary Biology, Faculty of Biology Ludwig‐Maximilians‐Universität München Munich Bavaria Germany; ^2^ Institute for Evolution and Biodiversity University of Münster Münster North Rhine‐Westphalia Germany

**Keywords:** de novo genes, gene expression, genome evolution, innovations, novelty, population genetics

## Abstract

*De novo* genes, which originate from noncoding DNA, are known to have a high rate of turnover over short evolutionary timescales, such as within a species. Thus, their expression is often lineage‐ or genetic background‐specific. However, little is known about their levels and breadth of expression as populations of a species diverge. In this study, we utilized publicly available RNA‐seq data to examine the expression of newly evolved open reading frames (neORFs) in comparison to non‐ and protein‐coding genes in *Drosophila melanogaster* populations from the derived species range in Europe and the ancestral range in sub‐Saharan Africa. Our datasets included two adult tissue types as well as whole bodies at two temperatures for both sexes and three larval/prepupal developmental stages in a single tissue and sex, which allowed us to examine neORF expression and divergence across multiple sample types as well as sex and population. We detected a relatively large proportion (approximately 50%) of annotated neORFs as expressed in the population samples, with neORFs often showing greater expression divergence between populations than non‐ or protein‐coding genes. However, differential expression of neORFs between populations tended to occur in a sample type‐specific manner. On the other hand, neORFs displayed less sex‐biased expression than the other two gene classes, with the majority of sex‐biased neORFs detected in whole bodies, which may be attributable to the presence of the gonads. We also found that neORFs shared among multiple lines in the original set of inbred lines in which they were first detected were more likely to be both expressed and differentially expressed in the new population samples, suggesting that neORFs at a higher frequency (i.e. present in more individuals) within a species are more likely to be functional.

## Graphical abstract

We show that newly‐evolved, expressed open reading frames (neORFs) identified in a set of inbred *Drosophila melanogaster* lines are also expressed in multiple tissues and developmental stages of pooled population samples from Europe and Africa, although differential expression of neORFs between populations is often tissue‐ or stage‐specific.

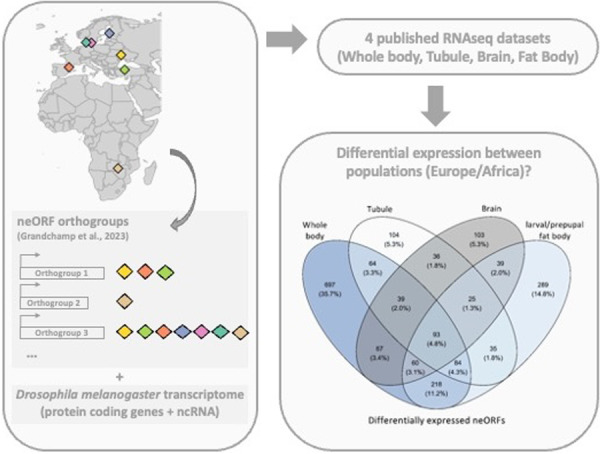

## 1 Introduction

It is well established that new genes can emerge from existing genes via duplication (Ohno 1970; Jacob 1977). More recently, an alternative mechanism has gained increasing attention, namely *de novo* gene emergence. *De novo* genes originate from noncoding DNA by acquiring the necessary features to become coding (Carvunis et al. 2012; Schlötterer 2015; Zhao et al. 2024). To be considered a *de novo* gene, a sequence needs to gain both transcription and an open reading frame (ORF) that, over time, might undergo further modification and be translated into a functional *de novo* protein (Schlötterer 2015; Schmitz and Bornberg‐Bauer 2017). The majority of *de novo* genes lack a known function. However, there are some examples, mainly of evolutionary older *de novo* genes (i.e. those shared across multiple species), where *de novo* genes perform an important function, such as influencing the fertility of *Drosophila* males (Lange et al. 2021) or being involved in cold resistance in codfish (Baalsrud et al. 2018). In contrast, studies focusing on shorter timescales (i.e. within a species or among very closely related species) often report a high turnover of *de novo* genes or their precursors (Neme and Tautz 2016; Grandchamp et al. 2023, 2024), indicating that many recently emerged *de novo* genes are eventually lost again. Thus, studying these shorter timescales is essential for understanding the dynamics of *de novo* gene birth (Blair et al. 2023; Grandchamp et al. 2023).

Grandchamp et al. (2023) examined the earliest stages of *de novo* gene formation by identifying expressed, newly‐evolved open reading frames (neORFs) in the genomes of seven inbred lines of *Drosophila melanogaster*, including one line from the species' ancestral range in sub‐Saharan Africa and six lines from the derived species range in Europe. This study found that neORFs were abundant in all lines (1,365–1,962 per line) but that typically one‐third of the neORFs were unique to a particular line and only 1% were present in all seven lines. This suggests that there is a high turnover of neORFs, with many neORFs arising within a population but being lost before they spread throughout the species. When comparing orthologous sequences and expression data between lines with an neORF and those lacking it, the most common difference was the gain or loss of expression (Grandchamp et al. 2023). That is, the ORF itself was present in multiple lines, but was expressed only in a subset of those lines, which was observed for about one‐half of polymorphic neORFs (i.e. those present in only a subset of lines). The next most common difference was the gain or loss of both expression and a complete ORF, which occurred for 35% of polymorphic neORFs. On the other hand, only about 15% of the polymorphic neORFs were expressed in multiple lines, but lacked a complete ORF in a subset of lines. Thus, one of the best indicators that an neORF was present and encoded a complete ORF in a particular genetic background was transcription, which only rarely occurred in the absence of an intact ORF.

Although the expression of *de novo* genes is enriched in males and/or male reproductive tissues (Zhao et al. 2014; Palmieri et al. 2014; Witt et al. 2019), little is known about the expression of recently emerged *de novo* genes across tissues, sexes, and developmental stages. Even less is known about variation in their expression among natural populations. To better understand the expression and coding potential (i.e. the presence of an intact ORF) of the neORFs annotated by Grandchamp et al. (2023), we reanalyzed RNA‐seq data from European and sub‐Saharan African *D. melanogaster* populations. European populations of *D. melanogaster* diverged from sub‐Saharan populations during the out‐of‐Africa expansion of the species about 10,000–15,000 years ago (Li and Stephan 2006; Sprengelmeyer et al. 2020) and modern‐day European populations show much greater genetic divergence with sub‐Saharan populations than they do with other European populations (Kapun et al. 2020, 2021). In general, European populations exhibit reduced genetic polymorphism in comparison to sub‐Saharan populations, which is thought to reflect both demography (a population bottleneck during colonization) and the effects of linked selection (selective sweeps) (Glinka et al. 2003; Li and Stephan 2006). However, there is also evidence that variation is maintained within non‐African populations by balancing selection (Bergland et al. 2014; Croze et al. 2017; Machado et al. 2021; Rudman et al. 2022; Glaser‐Schmitt et al. 2024). Gene expression has also diverged between African and non‐African populations (Meiklejohn et al. 2003; Catalán et al. 2012; Huylmans and Parsch 2014; Glaser‐Schmitt and Parsch 2023) and previous studies have found evidence that at least some of this divergence is adaptive (González et al. 2009; Mateo et al. 2014; Glaser‐Schmitt and Parsch 2018; Ramnarine et al. 2019; Glaser‐Schmitt and Parsch 2023). However, little is known about divergence in recently emerged neORF expression as populations diverge, especially in comparison to other types of genes.

To compare the patterns of neORF expression to other types of genes, we examined protein‐coding (PC), noncoding (ncRNA), and neORF gene expression. Similar to neORFs (Palmieri et al. 2014; Grandchamp et al. 2023), many ncRNAs (specifically long ncRNAs), have been shown to have line‐specific expression within *D. melanogaster* (Schor et al. 2018), suggesting that expression patterns of both ncRNAs and neORFs may differ from PC genes. In the current study, we examined expression in four RNA‐seq datasets comparing pooled population samples of European and sub‐Saharan *D. melanogaster* (Catalán et al. 2012; Huylmans and Parsch 2014; Glaser‐Schmitt and Parsch 2023; Voigt and Froschauer 2023). For three of the datasets that focused on adult flies, we also compared neORF expression between males and females, as sex is known to be have a strong influence on gene expression (Ellegren and Parsch 2007; Parsch and Ellegren 2013; Grath and Parsch 2016) and *de novo* genes often show enriched expression in males and/or male reproductive tissues (Zhao et al. 2014; Palmieri et al. 2014; Witt et al. 2019). We found that a relatively large proportion (typically around 50%) of the annotated neORFs are expressed in natural population samples and that they often show greater expression divergence between populations than protein‐coding genes or noncoding RNAs, although differential expression of neORFs between populations tended to occur in a sample type‐specific manner. The neORFs, however, exhibited less sex‐biased expression than genes of the other two classes. Moreover, neORFs that were at higher frequency among the initial lines used for annotation were more likely to be expressed in the new population samples and to differ significantly in expression level between populations.

## 2 Materials and Methods

### 2.1 Expression Data and Estimation

Raw RNA‐seq reads from pooled (8–12 lines per population) European and sub‐Saharan *D. melanogaster* population samples from adult male and female whole bodies maintained at 15°C and 28°C (Voigt and Froschauer 2023; France and Zambia, 3 biological replicates per sample), adult male and female Malpighian tubules (Huylmans and Parsch 2014; the Netherlands and Zimbabwe, 2 biological replicates per sample), adult male and female brains (Catalán et al. 2012; the Netherlands and Zimbabwe, 2 biological replicates per sample), and female larval (early and late wandering third instar) and white prepupal fat bodies (Glaser‐Schmitt and Parsch 2023; the Netherlands and Zambia, 3 biological replicates per sample) were downloaded from NCBI (BioProject accession numbers: PRJNA951064, PRJNA252932, PRJNA175361, and PRJNA905411, respectively). All of the examined RNA‐seq libraries were generated using polyA selection, with the exception of the larval and prepupal fat bodies, which were generated using ribosomal depletion. Reads were mapped to the *D. melanogaster* transcriptome, including the focal neORFs (Grandchamp et al. 2023) as well as mRNA, rRNAs, microRNAs, and noncoding RNAs with the FlyBase release version 6.55 annotation (Öztürk‐Çolak et al. 2024) using NextGenMap (Sedlazeck et al. 2013) in single‐ (Malpighian tubule and brain) or paired‐end (whole body and fat body) mode. Library sizes ranged from 22.0 to 94.5 million paired‐ or single‐end reads, 87.3%–98.1% of which could be mapped (Table S1).

In our expression analyses, we included all PC and NC genes as annotated in FlyBase release 6.55 (Öztürk‐Çolak et al. 2024) as well as neORFs annotated by Grandchamp et al. (2023). In each biological replicate, mapped read counts were summed over all transcripts of a gene so that expression analyses were carried out on a per gene basis. To categorize a gene as expressed in a sample, we calculated relative gene expression as transcripts per million (TPM; Wagner et al. 2013) for each sample type (i.e. biological replicates for each sample type were pooled). Following Grandchamp et al. (2023), a gene was categorized as expressed if TPM ≥ 0.5. Because most neORFs clustered in the same orthogroup were highly similar or identical (Grandchamp et al. 2023), we carried out expression and differential expression analyses of neORFs on a per orthogroup basis (i.e. counts for neORFs in the same orthogroup were summed; Data S1 and S2). For simplicity, these orthogroups are referred to throughout the text simply as ‘neORFs’ unless otherwise indicated.

### 2.2 Differential Expression Analysis

Differentially expressed genes were identified using a negative binomial test on gene count data (Data S1 and S2) as implemented in DESeq2 (Love et al. 2014) in R (R Core Team 2022) with a 5% false discovery rate. The independent filtering option was used to remove low count genes from the analysis, yielding 13,287–19,362 genes that could be included in the analysis, depending on the comparison (Table S2). Each sample type (whole body at 15°C, whole body at 28°C, adult Malpighian tubule, adult brain, and larval/prepupal fat body) was analyzed separately. For whole body, Malpighian tubule, and brain samples, we utilized a two‐factor model design with population and sex as factors. For larval/prepupal fat body samples, we used a one‐factor model design with a grouping variable consisting of six levels representing the six possible combinations of population and developmental stage, which allowed us to directly compare the African and European population for each examined stage without fitting a separate model for each stage. We tested for an over‐ or under‐representation of each gene type (PC, ncRNA, or neORF) among differentially expressed genes based on the proportion of differentially expressed genes among all analyzed genes for each sample type using a χ^2^ test. We used a *t*‐test to identify significant differences in the magnitude of the detected expression changes using the absolute value of the log_2_ fold changes (LFCs) for genes in each group as calculated by DESeq. 2 (Love et al. 2014). *p*‐values for both tests were adjusted with a Benjamini and Hochberg (1995; BH) multiple test correction.

### 2.3 Analysis of ORFs and Coding Potential

Because all of the RNA‐seq datasets were generated from pooled population samples, it is not possible to determine if any particular line in the sample contains the full‐length neORF, unless it can be covered by a single (or paired) RNA‐seq read generated from a single molecule. For this reason, to evaluate the coding potential of the analyzed neORFs, we followed a two‐step procedure to categorize the RNA‐seq read pairs mapping to an neORF. In the first step, we placed the reads into five categories (Data S3): complete ORF covered by single read pair (cat1), ORF partially covered by single read pair with no internal stop codon (cat2), internal stop codon detected (cat3), start codon not contained in read alignment despite being expected (cat4), or other (typically when the two paired reads mapped to different neORFs; cat5). In the second step, we performed a protein‐protein blast (blastp; Altschul et al. 1990) of the translated RNA‐seq alignment against the annotated neORF protein sequences and categorized read pairs into five additional categories based on their ORF coverage category and the *e*‐value of a blastp match to the expected orthogroup (Data S3): cat1 and *e* ≤ 1^−10^ (catA; full ORF and protein); cat2, *e* ≤ 1^−10^, and start codon present (catB; partial ORF and in‐frame protein); cat2, *e* ≤ 1^−10^, and start codon not present (catC; partial ORF and protein with frame unknown); cat2 and *e* > 1^−10^ (catD; partial ORF, but protein unclear); or cat1 and *e* > 1^−10^ (catE; full ORF but no protein match, that is, likely frameshift). To identify the number of neORFs for which we could confirm an intact ORF, we considered only neORFs that were expressed within a tissue type (TPM ≥ 0.5) that we could detect based on the read length (≤ 300 bp for larval/prepupal fat body and ≤ 200 bp for adult whole body). Because Malpighian tubule and brain reads were too short (50 bp single end; Table S1) to identify any full length neORFs (shortest neORF = 90 bp), we used the larval/pupal fat body data (L in Data S3) to identify full length neORFs, which allowed us to identify more neORFs than the adult whole body data (V in Data S3). An neORF was considered to be intact if at least one read was categorized into catA. We tested for an over‐ or under‐representation of intact neORFs among differentially expressed genes within each data set based on the proportion of intact neORFs among neORFs we could analyze for differential expression between sexes or populations (see Section 2.2) using a χ^2^ test.

To estimate the per‐site ratio of nonsynonymous‐to‐synonymous polymorphism (*p*_*N*_ */p*_*S*_) in neORFs, we considered the set of neORFs that had full‐length coding sequence coverage in the pooled European larval fat body RNA‐seq data set, which had the longest reads (150‐bp paired reads) and the most lines (12) included in the pool. To minimize sequencing and reverse‐transcription errors, we required a polymorphism to be present in two independent reads. Thus, a minimum of four full‐length reads was needed to detect a polymorphism. Numbers of nonsynonymous and synonymous sites were determined using the method of Nei and Gojobori (1986). A departure from a *p*_*N*_ */p*_*S*_ of 1.0, expected under no selective constraint, was tested using a χ^2^ goodness‐of‐fit test. Differences in *p*_*N*_ */p*_*S*_ between subsets of neORFs were tested using a χ^2^ test.

To identify protein coding genes in close proximity to the analyzed neORFs, we mapped the neORFs to the *D. melanogaster* reference genome (FlyBase release 6, GCF_000001215.4) using nucleotide blast [blastn; Altschul et al. 1990]) to identify their location in the reference genome. For each orthogroup the query neORF used to detect noncoding homologs in Grandchamp et al. (2023) was blasted against the genome and hits were required to have 100% coverage and an *e*‐value ≤ 0.01 to be considered a match for the neORFs location. In cases of multiple hits, the hit with the highest bitscore was considered the best match. Protein coding genes neighboring an neORF were identified using BEDtools (Quinlan and Hall 2010) window and FlyBase release 6.54 (Öztürk‐Çolak et al. 2024) to detect all genes overlapping with an neORF or within 1‐kb up‐ or down‐stream. To test if PC genes located in close proximity to differentially expressed neORFs were also differentially expressed, for each sample type we used a χ^2^ test to test for an over‐ or under‐representation of differentially expressed PC genes in close proximity to neORFs differentially expressed between populations in comparison to the expected proportion of differentially expressed PC genes within the respective sample type.

### 2.4 Analysis of neORF Frequency in the Original Lines Used for Annotation

To investigate the relationship between an neORF's expression and its frequency in the species, we combined our expression datasets (see Sections 2.1 and 2.2) with the orthogroup information from the original seven lines used for neORF annotation by Grandchamp et al. (2023). We determined the proportion of expressed neORFs (TPM ≥ 0.5) in relation to the number of lines in which the neORFs were present in the original data set. We merged the respective samples within each sample type by checking whether a given neORF was expressed with a TPM ≥ 0.5 in any of the samples. If it passed this threshold in at least one sample, that neORF was considered to be expressed in that sample type. Similarly, we determined the relationship between the frequency of the neORFs in the seven lines analyzed by Grandchamp et al. (2023) and the proportion of neORFs that were differentially expressed between the European and African populations (or the two sexes) in each sample type. We tested for an over‐ or under‐representation of expressed or differentially‐expressed neORFs in each frequency class (1–7 lines) using a χ^2^ test with a BH correction (Benjamini and Hochberg 1995).

## 3 Results

### 3.1 Expression of neORFs in Whole Adult Flies

To understand the expression and divergence of neORFs in comparison to other types of genes as populations diverge, we examined protein coding, noncoding, and neORF gene expression in pooled samples of whole adult *D. melanogaster* from a derived European and an ancestral African population. Depending on the sample, we detected expression of 45%–50% of the neORFs annotated by Grandchamp et al. (2023), which was lower than the percentage of PC genes (74%–92%), but comparable to that of ncRNAs (27%–76%). Within all gene types, similar numbers of genes were expressed at the two examined temperatures and in the European and African populations (Table 1). For all three types of genes, a greater proportion was detected as expressed in males than in females, although the difference between sexes was less pronounced for neORFs than for the other gene types (Table 1).

**Table 1:** Numbers of expressed (TPM ≥ 0.5) protein coding genes, ncRNAs, and neORFs in whole, adult flies.

Population	Sex	Temp	PC genes (%)	ncRNAs (%)	neORFs (%)
Eur	M	15	12,780 (91.4%)	1825 (71.4%)	3047 (53.6%)
Afr	M	15	12,854 (91.9%)	1823 (71.3%)	3129 (55.0%)
Eur	F	15	10,761 (76.9%)	825 (32.3%)	2752 (48.4%)
Afr	F	15	10,874 (77.7%)	849 (33.2%)	2772 (48.7%)
Eur	M	28	12,775 (91.3%)	1945 (76.1%)	3072 (54.0%)
Afr	M	28	12,828 (91.7%)	1913 (74.8%)	3105 (54.6%)
Eur	F	28	10,657 (76.2%)	749 (29.3%)	2598 (45.7%)
Afr	F	28	10,360 (74.1%)	706 (27.6%)	2604 (45.8%)

*Note:* Percentages are based on a total of 13,986 PC genes, 2557 ncRNAs, and 5687 neORFs for European (Eur) and African (Afr) populations at two temperatures (temp, °C).

**Figure 1:**
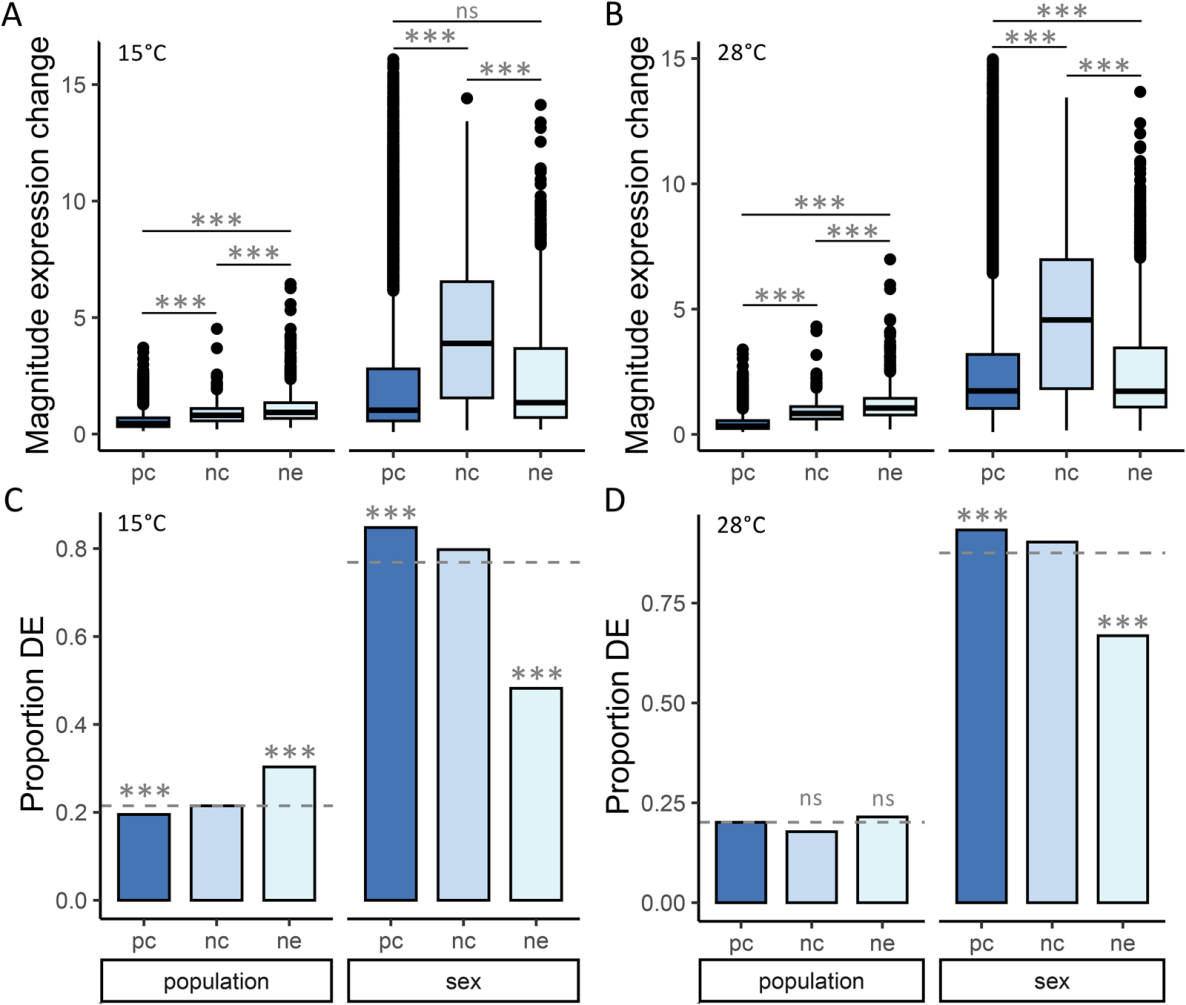
Differentially expressed (DE) genes in adult whole body. Shown are the (A, B) magnitude of expression changes (i.e. absolute value of the LFC, see Methods) for DE genes and the (C, D) proportion of DE (FDR < 0.05) protein coding genes (pc), ncRNAs (nc), and neORFs (ne) between populations or sexes at (A, C) 15°C or (B, D) 28°C. Boxes at the bottom of the figure indicate if a comparison is between populations (left in each panel) or between sexes (right in each panel). (A, B) Significance was assessed with a *t*‐test. (C, D) Dashed lines indicate the expected proportion of DE genes based on the total number of DE genes among all analyzed genes. Significance was assessed with a χ^2^ test. ***BH‐corrected *p* < 0.001, **p < 0.01, *p < 0.05, ns not significant after multiple test correction. *p* > 0.05 before multiple test correction not shown.

For all of the expressed genes in each category, we tested for differences in expression between sexes or populations (Table S2). When we tested for an over‐ or under‐representation of each gene type among differentially expressed genes (see Materials and Methods for more details), at 15°C, we detected an excess of neORFs differentially expressed between populations, but a paucity of PC genes (χ^2^ test; BH‐corrected *p* < 10^−15^ for both; Figure 1C). In contrast, we detected a paucity of neORFs differentially expressed between sexes, but an excess of PC genes (χ^2^ test; BH‐corrected *p* < 10^−15^ for both; Figure 1C). At 28°C, we detected a similar pattern for sex‐biased genes (χ^2^ test; BH‐corrected *p* < 10^−15^ for both; Figure 1D), but not population‐biased genes (χ^2^ test; BH‐corrected *p* > 0.05 for both; Figure 1D). When we examined the magnitude of expression changes (i.e. the absolute value of the LFC) for differentially expressed genes, we detected significant differences among all gene types for both sex‐ and population‐biased genes at both temperatures (*t*‐test; BH‐corrected *p* < 10^−15^ for all; Figure 1A–B), with the exception of sex‐biased PC genes versus neORFs at 15°C, which was no longer significant after multiple test correction (*t*‐test; BH‐corrected *p* = 0.0591; Figure 1A). In general, expression changes of differentially expressed neORFs and ncRNAs were larger than for PC genes, while expression changes in neORFs were larger than in ncRNAs for population‐biased genes but smaller for sex‐biased genes (Figure 1A–B). Thus, in whole adult flies, we detected differences in the magnitude and proportion of population‐ and sex‐biased genes in neORFs in comparison to other types of genes, suggesting that the evolutionary forces affecting neORF expression as populations diverge may differ from other types of genes.

### 3.2 Expression of neORFs in Adult Malpighian Tubule and Brain

To characterize the expression and expression divergence of neORFs in different tissues as populations diverge, we examined RNA‐seq data from Malpighian tubules and brains of adult *D. melanogaster* from a European and an African population. Similar to the observations for whole flies, approximately 52%–62% of the neORFs were expressed in each tissue, which was less than the percentage of PC genes (64%–72%), but greater than that of ncRNAs (28%–44%). For each gene type, similar numbers of genes were expressed in each population with the exception of neORFs and ncRNAs in the female brain, where we detected ~5%–6% fewer in the European population (Table 2). Unlike in whole flies, there were not large differences in the number of expressed genes of any category between the sexes (Table 2).

**Table 2:** Numbers of expressed (TPM ≥ 0.5) protein coding genes, ncRNAs, and neORFs in adult Malpighian tubule and brain.

Population	Sex	Tissue	PC genes (%)	ncRNAs (%)	neORFs (%)
Eur	M	Tubule	9668 (69.1%)	752 (29.4%)	3033 (53.3%)
Afr	M	Tubule	9339 (66.8%)	725 (28.4%)	3009 (52.9%)
Eur	F	Tubule	9109 (65.1%)	724 (28.3%)	3004 (52.8%)
Afr	F	Tubule	9019 (64.5%)	723 (28.3%)	3005 (52.8%)
Eur	M	Brain	10,008 (71.6%)	1048 (41.0%)	3396 (59.7%)
Afr	M	Brain	10,115 (72.3%)	1034 (40.4%)	3329 (58.5%)
Eur	F	Brain	9918 (70.9%)	983 (38.4%)	3259 (57.3%)
Afr	F	Brain	10,144 (72.5%)	1137 (44.5%)	3531 (62.1%)

*Note:* Percentages are based on a total of 13,986 PC genes, 2557 ncRNAs, and 5687 neORFs for European (Eur) and African (Afr) populations in the brain and Malpighian tubule (tubule).

**Figure 2:**
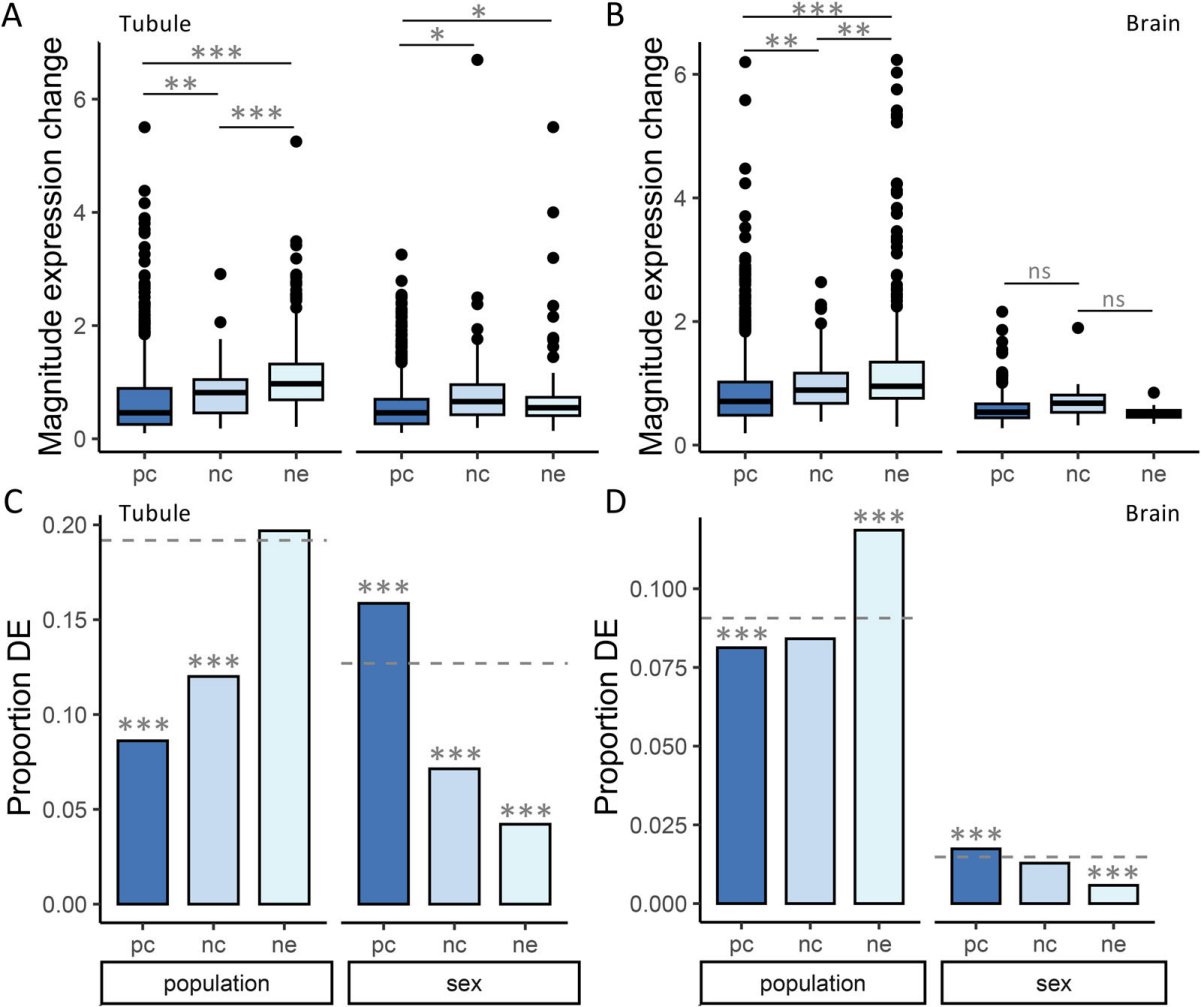
Differentially expressed (DE) genes in somatic tissues. Shown are the (A, B) magnitude of expression changes (i.e. absolute value of the LFC, see Methods) for DE genes and the (C, D) proportion of DE (FDR < 0.05) protein coding genes (pc), ncRNAs (nc), and neORFs (ne) between populations or sexes in the (A, C) Malpighian tubule or (B, D) brain. Boxes at the bottom of the figure indicate if a comparison is between populations (left in each panel) or between sexes (right in each panel). (A, B) Significance was assessed with *t*‐test. (C, D) Dashed lines indicate the expected proportion of DE genes based on the total number of DE genes among all analyzed genes. Significance was assessed with a χ^2^ test. ***BH‐corrected *p* < 0.001, **p < 0.01, *p < 0.05, ns not significant after multiple test correction. *p* > 0.05 before multiple test correction not shown.

We identified differentially expressed genes between sexes or between populations (Table S2) for both tissues and tested for an over‐ or under‐representation of each gene type among differentially expressed genes (see Section 2 for more details). In the Malpighian tubule, we detected a large paucity of protein and noncoding genes differentially expressed between populations (χ^2^ test; BH‐corrected *p* < 10^−5^ for both), but the expected proportion of differentially expressed neORFs (Figure 2C). On the other hand, we detected a paucity of ncRNAs and neORFs differentially expressed between sexes, but an excess of PC genes (χ^2^ test; BH‐corrected *p* < 10^−5^ for all; Figure 2C). In the brain, we detected a similar pattern to whole body at 15°C (Figure 1C), with an excess of neORFs but a paucity of PC genes differentially expressed between populations (χ^2^ test; BH‐corrected *p* < 10^−7^ for both; Figure 2D), and a reciprocal paucity of neORFs but excess of PC genes differentially expressed between sexes, (χ^2^ test; BH‐corrected *p* < 10^−4^ for both; Figure 2D). Thus, similar to whole flies, in comparison to PC genes neORFs in somatic tissues tended to be over‐represented among genes differentially expressed between populations but underrepresented among genes differentially expressed between sexes, further underscoring that the evolutionary forces that affect their expression may differ from other types of genes.

When we examined the magnitude of expression changes for differentially expressed genes, we detected significant differences among all gene types for population‐biased genes in both tissues (*t*‐test; BH‐corrected *p* < 0.006 for all; Figure 2A,B), with expression changes largest for neORFs, followed by ncRNAs and then PC genes (Figure 2A,B). On the other hand, among sex‐biased genes, only neORFs and ncRNAs in the Malpighian tubule showed significantly larger expression changes than PC genes (*t*‐test; BH‐corrected *p* < 0.020 for both; Figure 2A). Thus, in somatic tissues expression changes of differentially expressed neORFs and ncRNAs were larger than for PC genes, with the exception of sex‐biased genes in the brain, while expression changes in neORFs were larger than in ncRNAs for population‐biased genes.

### 3.3 Expression of neORFs in Larval/Prepupal Fat Body

To better understand the expression and expression divergence of neORFs in a single tissue during larval development as populations diverge, we examined their expression in comparison to PC genes and ncRNAs in pooled samples of fat bodies from early or late wandering third instar or prepupal *D. melanogaster* from a European and an African population. Unlike in adult flies, the number of PC and noncoding genes expressed differed by more than 10% between populations for early and late wandering stages, while the number of neORFs expressed differed by 7.5% between populations specifically during the late wandering larval stage (Table 3). Thus, the proportion of expressed genes was dependent upon both population and developmental stage.

**Table 3:** Numbers of expressed (TPM ≥ 0.5) protein coding genes, ncRNAs, and neORFs in larval/prepupal fat body.

Population	Stage	PC genes (%)	ncRNAs (%)	neORFs (%)
Eur	Early	9556 (68.3%)	865 (33.8%)	2435 (42.8%)
Afr	Early	11,139 (79.6%)	1142 (44.7%)	2611 (45.9%)
Eur	Late	12,256 (87.6%)	1656 (64.8%)	2969 (52.2%)
Afr	Late	10,092 (72.2%)	898 (35.1%)	2543 (44.7%)
Eur	Prepup	11,509 (82.3%)	1363 (53.3%)	2816 (49.5%)
Afr	Prepup	11,609 (83.0%)	1393 (54.5%)	2840 (49.9%)

*Note:* Percentages are based on a total of 13,986 PC genes, 2557 ncRNAs, and 5687 neORFs for European (Eur) and African (Afr) populations in the early and late wandering and prepupal (prepup) stages.

**Figure 3:**
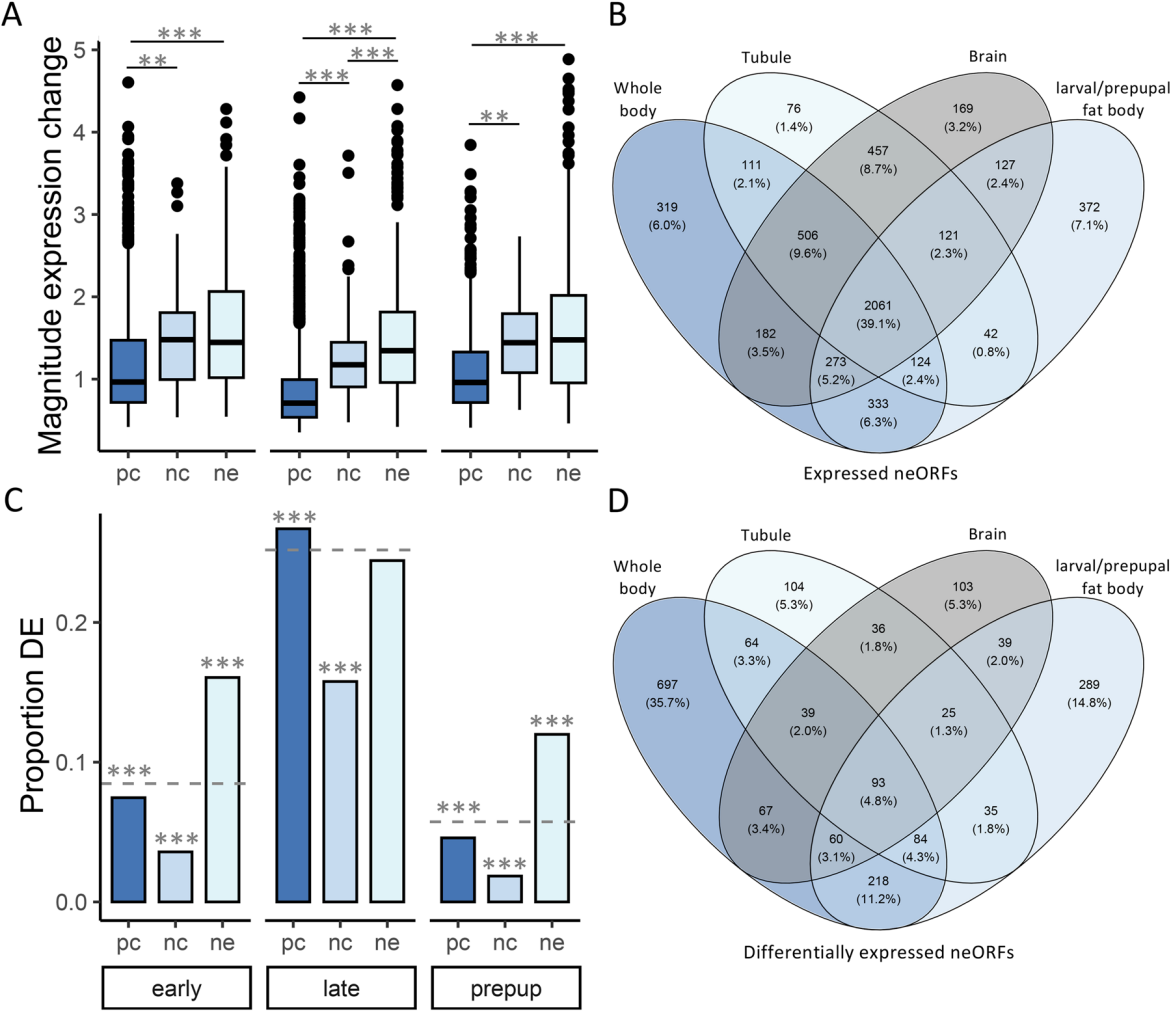
Differentially expressed (DE) genes in the larval/prepupal fat body and overlapping neORFs among datasets. Shown are the (A) magnitude of expression changes for DE genes and the (C) proportion of DE (FDR < 0.05) protein coding genes (pc), ncRNAs (nc), and neORFs (ne) between populations in the early and late wandering larval or prepupal (prepup) stages. Boxes at the bottom of the figure indicate the stage of a population comparison. (A) Significance was assessed with a *t*‐test. (C) Dashed lines indicate the expected proportion of DE genes based on the total number of DE genes among all analyzed genes. Significance was assessed with a χ^2^ test. ***BH‐corrected *p* < 0.001, **p < 0.01, *p < 0.05, ns not significant after multiple test correction. *p* > 0.05 before multiple test correction not shown. Shown are the number and percentage of (B) expressed and (D) differentially expressed neORFs between populations in all examined sample types.

To better understand expression divergence of neORFs in comparison to other types of genes during development, we identified differentially expressed genes between populations (Table S2) and tested for an over‐ or under‐representation of each gene type among differentially expressed genes (see Materials and Methods for more details) for each examined developmental stage. We detected a paucity of ncRNAs among differentially expressed genes for all stages as well as a paucity of PC genes in the early wandering and prepupal stages, but an excess in the late wandering stage (χ^2^ test; BH‐corrected *p* < 10^−9^ for all; Figure 3D). Somewhat reciprocally, in the early wandering and prepupal stages, we detected an excess of differentially expressed neORFs (χ^2^ test; BH‐corrected *p* < 10^−14^ for both; Figure 3D), which was similar to the general patterns of differential neORF and PC gene expression that we observed in adults (Figures 1 and 2). When we examined the magnitude of expression changes for differentially expressed genes, expression changes in neORFs and ncRNAs were significantly larger than for PC genes for all examined stages (*t*‐test; BH‐corrected *p* < 0.004 for all; Figure 3A), with changes in neORFs being significantly larger than ncRNA genes specifically during the late wandering stage (*t*‐test; BH‐corrected *p* = 1.23 × 10^−12^; Figure 3A).

### 3.4 neORF Expression Overlap Across All Datasets

To better understand how broadly expressed the examined neORFs were across the examined datasets, we compared the number of neORFs expressed or differentially expressed across the examined sample types. We detected 39.1% of expressed neORFs as expressed in all sample types, while only 1.4%–7.1% were detected as expressed in a single sample type (Figure 3B). On the other hand, neORFs detected as differentially expressed between populations tended to be more sample type‐specific, with only 4.8% of neORFs detected as population‐biased in all sample types, while 5.3%–35.7% were detected as population‐biased in a single sample type (Figure 3D). When we examined sex‐bias in samples for which we could analyze both sexes, the majority of sex‐biased neORFs were detected exclusively in whole body samples (2,739 neORFs; 95%), another eight (0.3%) of which were detected in all examined sample types and another four (0.1%) or 99 (3.4%) of which were detected in only whole bodies and brain or Malpighian tubule, respectively (Figure S1). Fewer neORFs were detected as sex‐biased in the brain or Malpighian tubule, 4–29 (0.1%–1.0%) of which were detected exclusively in one of these tissues (Table S2, Figure S1). Thus, neORFs tended to be expressed across multiple sample types, but differential expression tended to occur in a more sample type‐specific manner.

### 3.5 Confirmation of Intact neORFs

Although transcription of an neORF is the best indicator of the presence of an neORF in a particular genetic background (Grandchamp et al. 2023), we further confirmed that the examined neORFs also had an intact open reading frame in the examined datasets. To detect complete, intact ORFs, we assessed mapped reads for complete ORFs covered by a single read pair that matched an neORF in the corresponding orthogroup (see Section 2.3). Only neORFs that could be detected with this method were included in the analysis (i.e. those with a length of 300 bp or 200 bp or shorter, depending on the examined sample type). With this conservative approach, we detected 31.1%–43.9% of neORFs as intact in each population in each sample type (Table S3). We detected slightly more neORFs (0.4%–2.2%) as intact in European than in African populations for all sample types except in the brain, where similar numbers were detected. Within each examined tissue type, 36.4%–51.4% of neORFs in total could be detected as intact, while only 25.9%–37.2% could be detected in both populations (Table S3). Thus, 9.9%–15.6% of analyzed neORFs could be detected as intact in only one population. Among differentially expressed neORFs, we detected more intact neORFs than expected by chance among genes differentially expressed between populations (χ^2^ test; *p* < 10^−8^ for all; Table S3) for all sample types except the Malpighian tubule, but approximately the expected number of intact neORFs for genes differentially expressed between sexes (*p* > 0.08 for all; Table S3). Thus, although we could only confirm an intact ORF for approximately half of the analyzed neORFs, they were enriched among differentially expressed genes, suggesting that neORFs that contribute to expression divergence between populations are more likely to have intact ORFs and may play a functional role that has facilitated their differentiation in expression.

### 3.6 Relationship Between the Expression of neORFs and Their Frequency in the Original Lines Used for Annotation

**Figure 4:**
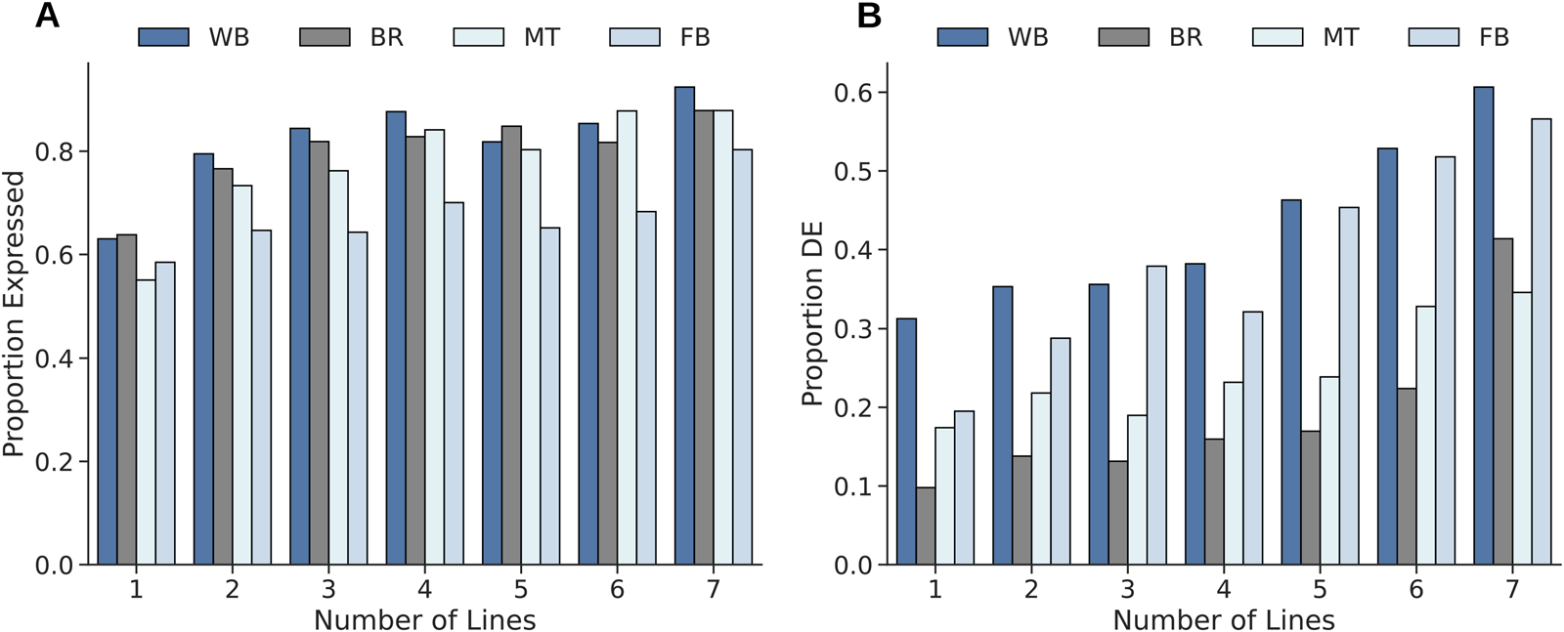
Expression of neORFs in the whole body (WB), brain (BR), Malpighian tubule (MT), and larval/prepupal fat body (FB), and their frequency in the initial lines used for annotation. (A) Proportion of neORFs expressed in each sample type binned by the number of lines in which the respective neORF was present in the original data set (Grandchamp et al. 2023). (B) Proportion of differentially expressed neORFs between populations in relation to the number of lines containing the neORF in the original data set.

To estimate the frequency of neORFs within the species, we determined the number of lines in which each neORF was present in the original detection set of seven lines in Grandchamp et al. (2023). First, we examined whether the neORFs that were present in more lines in the original data set were expressed at higher proportions in the examined datasets. The highest proportion of expressed neORFs in the examined datasets was for those present in all seven lines in the original data set (Figure 4A, Data S4), ranging from 80% in larval/prepupal fat body to 92% in whole body. In contrast, a much lower proportion of the neORFs present in a single line in the original data set were expressed in the examined datasets, ranging from 55% (Malpighian tubule) to 63% (brain).

Next, we investigated whether the number of lines containing an neORF in the original data set was associated with differential expression between populations in the examined datasets (Figure 4B, Data S4). In general, the proportion of neORFs differentially expressed between populations increased with the number of lines containing the neORF in the original data set, with neORFs detected in a single line having the lowest proportion of differentially expressed neORFs (8%–31% in brain and whole body, respectively) and neORFs detected in all seven lines the highest (33%–61% in Malpighian tubule and whole body, respectively; Figure 4B). For example, in the whole body, 31% of single‐line neORFs were differentially expressed between populations, which increased to 38% for neORFs present in four lines, and 61% for neORFs present in seven lines (Figure 4B). Indeed, for all sample types neORFs present in one line in the original data set were less likely to be differentially expressed than expected (χ^2^ test; BH‐corrected *p* < 0.006 for all), while the opposite was true for neORFs present in six lines (χ^2^ test; BH‐corrected *p* < 0.05 for all except the Malpighian tubule) or seven lines (χ^2^ test; BH‐corrected *p* < 0.04 for all). Thus, neORFs that were more widespread (i.e. expressed in more lines) in the original detection set were more often differentially expressed between populations.

For the expression datasets that included both sexes, we examined the proportion of neORFs differentially expressed between sexes in relation to their frequency in the original data set (Figure S2, Data S4). In whole body samples, we observed a similar pattern to that observed for population‐biased genes, with the proportion of sex‐biased neORFs increasing as the number of lines containing the neORF in the original data set increased, (71% for single‐line neORFs *vs.* 76% and 93% for neORFs present in four and seven lines, respectively; Figure S2). In whole bodies, we detected an excess of sex‐biased neORFs among those present in all lines (χ^2^ test; BH‐corrected *p* < 0.001) and an under‐representation of sex‐biased neORFs among those present in a single line (χ^2^ test; BH‐corrected *p* < 0.01), but approximately the expected number for the remaining categories (2–5 lines containing an neORF). In the brain, we detected very few sex‐biased neORFs (Table S2); thus, unsurprisingly, sex‐biased neORFs were not significantly over‐ or underrepresented for any of the categories (*p* = 1 for all). In the Malpighian tubule, we only detected neORFs present in a single line in the original data set as having fewer sex‐biased neORFs than expected (χ^2^ test; BH‐corrected *p* < 0.05). Thus, while we found some evidence that widespread neORFs were more likely to be sex‐biased, this pattern was less robust than that detected for population‐biased neORFs.

### 3.7 Selective Constraint on neORFs and Differential Expression in Neighboring Genes

To test for selective constraint on the amino acid sequence of neORFs, we examined the per‐site ratio of nonsynonymous‐to‐synonymous polymorphism (*p*_*N*_ */p*_*S*_) for all neORFs that had full‐length coverage in at least four RNA‐seq reads in the pooled European larval fat body data set, which had the longest reads and the largest number of lines in the pool. For the 1033 neORFs meeting these criteria, the combined *p*_*N*_ */p*_*S*_ was 0.70, which was significantly less than 1.0 (χ^2^‐test, *p* < 0.0001), indicating purifying selection against a proportion of nonsynonymous mutations. Given their short lengths and small number of polymorphic sites (Data S5), it was not possible to test for selection acting on individual neORFs. However, when comparing neORFs present in only a single line in the original set of lines used for annotation to those present in multiple lines, there was not a significant difference in *p*_*N*_ */p*_*S*_ (χ^2^‐test, *p* > 0.5; Table S4). Thus, there was no evidence for greater selective constraint on the latter set of neORFs.

In order determine if protein coding genes neighboring differentially expressed neORFs were also differentially expressed, we identified PC genes in close proximity to neORFs differentially expressed between European and African populations (see Materials and Methods). For all sample types except whole bodies at 28°C, we detected significantly more differentially expressed PC genes neighboring differentially expressed neORFs than expected by chance (χ^2^‐test, *p* < 1.8 × 10^‐4^ for all; Table 4). This association with differential expression in neighboring PC genes suggests that there may be some shared regulatory basis underlying the detected changes in neORF and PC gene expression between populations. The excess of nearby differentially expressed PC genes we detected was particularly high in the early larval and prepupal fat body and Malpighian tubule, where 4.5–6.5‐fold more PC genes than expected were detected as differentially expressed (Table 4), suggesting that in these tissues the transcription of neORFs may be more tightly linked to the regulation of neighboring genes than in the other examined sample types.

**Table 4:** Differential expression of protein coding genes neighboring differentially expressed neORFs.

Sample type	Num	DE	Exp DE	*p* value
Whole body 15°C	2352	581	459.02	2.20E‐10
Whole body 28°C	1760	349	354.05	0.764
Early larval fat body	590	213	43.99	< 2.2E‐16
Late larval fat body	1081	534	288.63	< 2.2E‐16
Prepupal fat body	453	135	20.75	< 2.2E‐16
Malpighian tubule	916	353	78.90	< 2.2E‐16
Brain	809	95	65.75	1.70E‐04

*Note:* The total number (num) of PC genes located within 1‐kb up‐ or downstream of a differentially expressed (DE) neORF, the number of which were DE in the same sample type, and the number expected (exp) to be DE based on the proportion of PC genes DE between populations in the respective tissue type. *p*‐values are from a χ2 test of the observed DE PC genes in comparison to expectations for that tissue type.

## 4 Discussion

In this study, we used published RNA‐seq data to characterize the expression of neORFs in derived (European) and ancestral (African) populations of *D. melanogaster* across multiple sample types as these populations diverged. We found that the neORFs that were originally annotated in a sample of seven inbred lines (six from European populations and one from an African population; Grandchamp et al. 2023) showed widespread expression in the examined population samples, with typically 50% or more of the annotated neORFs detected as expressed (Tables 1, 2, 3), although the number of detected neORFs varied depending upon sample type as well as, in some cases, sex, population, or developmental stage. Indeed, the percentage of expressed neORFs in each data set was often as high or higher than that for ncRNAs, which may be attributable to the high tissue‐ and developmental‐specificity that has been observed for many ncRNAs (Necsulea et al. 2014; Chen et al. 2016), but was lower than for PC genes (Tables 1, 2, 3). We also detected differences in the number of expressed neORFs between populations specifically in female brains and late wandering larvae (Tables 2 and 3), suggesting differences in the total number of expressed neORFs between populations may occur in a context‐specific manner over short evolutionary timescales.

The detected neORFs tended to be fairly broadly expressed, with the majority detected across multiple sample/tissue types (Figure 3B); however, neORFs detected as differentially expressed tended to occur in a more context‐specific manner (Figures 3D and S1). In particular, sex‐biased neORFs were almost exclusively detected in whole, adult flies (Figure S1), where we also detected a general pattern of higher expression in males (i.e. a higher proportion of all gene types, including neORFs, were expressed in males than in females; Table 1), which may have been driven by the presence of the gonads, which were not present in any of the other examined tissues. Previous studies have found that expression of *de novo* genes is often enriched in males and/or male reproductive tissues (Zhao et al. 2014; Palmieri et al. 2014; Witt et al. 2019). Indeed, with the exception of whole‐body samples, which included gonads, we detected fewer neORFs as differentially expressed between sexes than between populations (Table S2), suggesting that sex‐bias in neORFs may be largely restricted to the gonads.

This potential restriction of sex‐bias in neORFs to the gonads is underscored by our findings that neORFs were over‐represented among genes differentially expressed between populations, but underrepresented among genes differentially expressed between sexes, while protein coding genes showed the opposite pattern and ncRNAs were underrepresented for both (Figures 1, 2, 3). Thus, over short evolutionary timescales, neORFs were often expressed at different levels between European and African populations, suggesting that they may play an important role during adaptation and population differentiation. This contribution also appears to be context‐specific (Figure 3D), as has previously been found for ncRNA and PC genes (Glaser‐Schmitt and Parsch 2023). However, with pooled population data we could not distinguish between changes in the expression level of an neORF that was already established in the species versus changes in the pervasiveness of an neORF's expression among individuals within a population. In other words, we cannot distinguish between neORFs that are expressed in all (or a similar number of) lines but at different levels in each population and neORFs that are polymorphic for expression within the populations but are expressed in more lines in one population than the other. This latter scenario is likely to be more common, given that previous studies have found that over shorter evolutionary distances, i.e. within species or between closely related species, *de novo* genes have high rates of turnover (Neme and Tautz 2016; Schmitz et al. 2018; Grandchamp et al. 2023, 2024) and the vast majority of *D. melanogaster* neORFs are polymorphic in expression among lines with the gain/loss of expression being the most common difference between lines containing an neORF and those lacking it (Grandchamp et al. 2023). However, our finding that neORFs differentially expressed between populations tended to be sample type‐specific (Figure 3D) suggests that within species this turnover may occur in a tissue‐specific manner, rather than across all tissues simultaneously. Indeed, previous studies similarly found that *de novo* gene expression is often tissue‐specific (Schmitz et al. 2020; Peng and Zhao 2024). Expression changes in differentially expressed genes also tended to be of larger magnitude for neORFs and ncRNAs than PC genes, with neORF expression changes often larger than those for ncRNAs (Figures 1, 2, 3), suggesting that neORF expression may be less constrained than that of PC and ncRNAs, which would also be in line with a high turnover of *de novo* genes.

These differences that we detected in the magnitude and proportion of neORFs differentially expressed between populations and sexes in comparison to other types of genes (Figures 1, 2, 3) also suggest that at least some of the analyzed neORFs have acquired novel regulatory mechanisms that have facilitated context‐specific changes in gene expression (Figure 3D and S1) between sexes and as European and African populations diverged. On the other hand, we found that PC genes in close proximity to differentially expressed neORFs were also more likely to be differentially expressed (Table 4), which suggests that many neORFs also to some degree share underlying regulatory mechanisms with nearby genes. Thus, over short evolutionary scales, changes, including context‐specific changes, in neORF expression can be driven by changes in both novelly acquired and co‐opted regulatory elements. However, the overall degree to which each type of regulation contributes to the evolution of neORF expression remains unknown.

Using *p*_*N*_ */p*_*S*_ as a measure of selective constraint, we detected potential purifying selection on the examined neORFs, suggesting that at least a portion of the examined neORFs are being maintained by selection in *D. melanogaster*. However, we could not detect any differences in the degree of selection between neORFs originally detected in a single line versus those present in multiple lines (Table S4), although this finding may result from a lack of power due to the limited number of neORFs we could include in these analyses. Overall, we found that neORFs that were more widespread among the original, annotated lines (Grandchamp et al. 2023) were more likely to be expressed in the examined datasets as well as to differ significantly in expression between populations (Figure 4). These findings suggest that some of the widespread neORFs were able to establish within the species, integrating into the transcriptional program and potentially playing a functional role, which may have facilitated their rapid differentiation in expression between populations. Although some of these neORFs may have established within *D. melanogaster* and play a functional role, this role is unlikely to be essential. This further suggests that their expression is likely less constrained than ncRNAs, which can play crucial regulatory roles (Bunch 2018; Panni et al. 2020), or PC genes and is more able to diverge between populations. Nevertheless, in all of the examined sample types we were able to detect the expression of more than half of the neORFs present in only a single line in the original data set (Figure 4A; Grandchamp et al. 2023), suggesting that some of them may be more widespread than previously assumed, especially when more tissues or developmental stages are considered.

There are some limitations to using RNA‐seq data from pooled population samples, such as the inability to distinguish between changes in expression level versus differences in the number of lines expressing the neORF within a population, which we discussed above. Another limitation to the use of pooled RNA‐seq data is that we can only be certain that the full‐length coding sequence of an neORF is present in at least one line in the population if the complete coding sequence is covered by a single (or paired) RNA‐seq read generated from a single molecule. For the larval fat body data set (paired‐reads of 150 bp) the longest ORF that could be detected was 300 bp, which was not too severe of a limitation, as 83.2% of the annotated neORFs have a length of 300 bp or less. However, for the whole fly (paired reads of 100 bp) the maximum length we could detect was 200 bp, which covered only 58.1% of the annotated neORFs. For the brain and Malpighian tubule datasets (single reads of 50 bp), the read length was too short to detect any full‐length coding sequences, as the shortest neORF length was 90 bp. For these tissues we used the larval fat body data set to confirm intact ORFs. We were able to confirm 36.4%–51.4% of analyzed neORFs as intact, which is likely an underestimate of the true number of intact neORFs given their relatively low expression (Data S1 and S2) and our conservative requirement that an ORF be confirmed within a single read pair. Moreover, the neORFs that we could confirm as intact were enriched among differentially expressed genes, suggesting that these neORFs may be functional and contribute to differences between populations and/or sexes.

## 5 Conclusions

We found that approximately half of the neORFs identified by Grandchamp et al. (2023) in an original sample of seven inbred lines were broadly expressed across tissues in larger, pooled samples from natural *D. melanogaster* populations. Our results are consistent with previous findings that neORFs have a high turnover rate over short evolutionary timescales (Neme and Tautz 2016; Grandchamp et al. 2023, 2024); however, our finding that neORF expression divergence between populations tended to be more sample type‐specific suggests that this turnover may be tissue‐dependent. We also detected many neORFs with widespread expression as differentially expressed between populations, suggesting that some of these neORFs have successfully established within *D. melanogaster* and quickly differentiated among populations. Overall, these results contribute to our understanding of *de novo* gene expression dynamics and turnover over short evolutionary timescales and provide insight into neORF expression specificity, establishment, and turnover.

## Conflicts of Interest

The authors declare no conflicts of interest.

## Supporting information


**Figure S1 Overlapping sex‐biased neORFs in the examined sample types.** Shown are the number and percentage of neORFs differentially expressed between sexes in each sample type.

**Figure S2 Proportion of differentially expressed neORFs between sexes in relation to their frequency in the original lines used for annotation.** Shown is the percentage of differentially expressed neORFs between sexes in whole body (WB), Malpighian tubule (MT), and brain (BR).

**Table S1 Library size and mapping efficiency for all samples**.

**Table S2: Analyzed and differentially expressed genes**.

**Table S3: Detection of intact neORFs**.

**Table S4: Number (num) of synonymous (S) versus nonsynonymous (N) polymorphisms in neORFs present in a single versus multiple lines in the original dataset**.

**Data S1 TPM and DESeq2 results for all datasets**.

**Data S2 Gene counts for each dataset**.

**Data S3 neORF categories for each dataset**.

**Data S4 Counts and statistics for the analysis of differential expression of neORFs based on their frequency in the original lines used for annotation**.

**Data S5 Frequency of neORFs in original lines used for annotation,** ***pN*** **/** ***pS*** **of analyzed neORFs, and protein coding genes neighboring neORFs**.
