# Supplementary material for: Expression of De Novo Open Reading Frames in Natural Populations of Drosophila melanogaster
Source: J Exp Zool B Mol Dev Evol. 2025 Apr 15;344(7):415–27. doi: 10.1002/jez.b.23297 (PMC12576379; doi:10.1002/jez.b.23297)
Supplement: Supplementary file 1 — Figure S1 Overlapping sex‐biased neORFs in the examined sample types. Shown are the number and percentage of neORFs differentially expressed between sexes in each sample type. [file JEZ-344-415-s006.pdf]

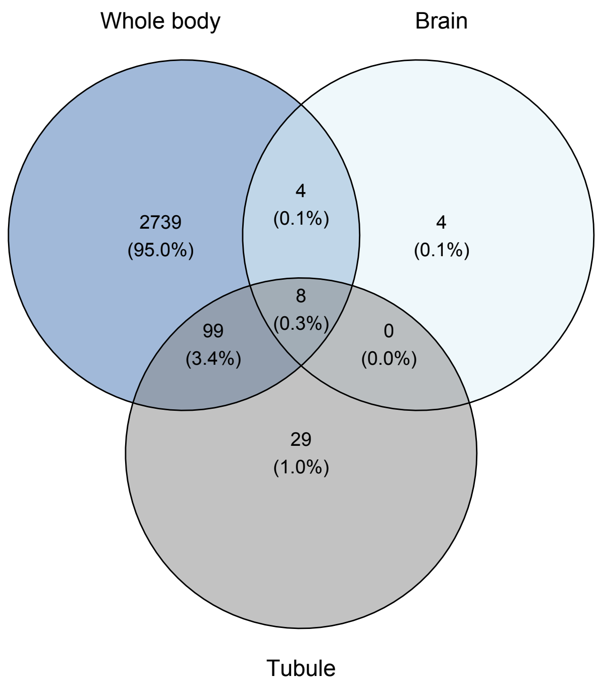

Figure S1 Overlapping sex-biased neORFs in the examined sample types. Shown are the number and percentage of neORFs differentially expressed between sexes in each sample type.
