# Supplementary material for: Expression of De Novo Open Reading Frames in Natural Populations of Drosophila melanogaster
Source: J Exp Zool B Mol Dev Evol. 2025 Apr 15;344(7):415–27. doi: 10.1002/jez.b.23297 (PMC12576379; doi:10.1002/jez.b.23297)
Supplement: Supplementary file 2 — Figure S2 Proportion of differentially expressed neORFs between sexes in relation to their frequency in the original lines used for annotation. Shown is the percentage of differentially expressed neORFs between sexes in whole body (WB), Malpighian tubule (MT), and brain (BR). [file JEZ-344-415-s011.pdf]

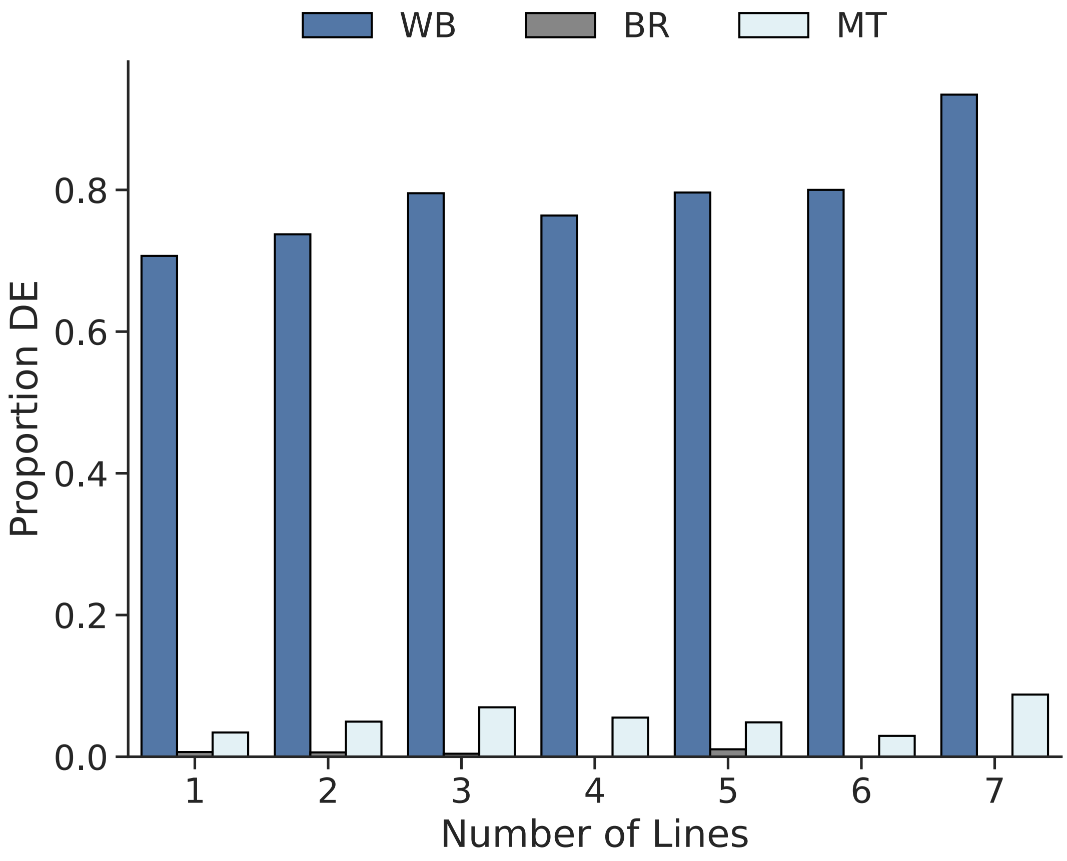

Figure S2 Proportion of differentially expressed neORFs between sexes in relation to their frequency in the original lines used for annotation. Shown is the percentage of differentially expressed neORFs between sexes in whole body (WB), Malpighian tubule (MT), and brain (BR).
